# Target Tracking and Classification from Labeled and Unlabeled Data in Wireless Sensor Networks

**DOI:** 10.3390/s141223871

**Published:** 2014-12-11

**Authors:** Jaehyun Yoo, Hyoun Jin Kim

**Affiliations:** Department of Mechanical and Aerospace Engineering, Seoul National University, 599 Gwanangno, Gwanak-gu, Seoul KS013, Korea; E-Mail: yjh5455@gmail.com

**Keywords:** low-cost sensor network, multi-target tracking, semi-supervised learning, Gaussian process

## Abstract

Tracking the locations and identities of moving targets in the surveillance area of wireless sensor networks is studied. In order to not rely on high-cost sensors that have been used in previous researches, we propose the integrated localization and classification based on semi-supervised learning, which uses both labeled and unlabeled data obtained from low-cost distributed sensor network. In our setting, labeled data are obtained by seismic and PIR sensors that contain information about the types of the targets. Unlabeled data are generated from the RF signal strength by applying Gaussian process, which represents the probability of predicted target locations. Finally, by using classified unlabeled data produced by semi-supervised learning, identities and locations of multiple targets are estimated. In addition, we consider a case when the labeled data are absent, which can happen due to fault or lack of the deployed sensor nodes and communication failure. We overcome this situation by defining artificial labeled data utilizing characteristics of support vector machine, which provides information on the importance of each training data point. Experimental results demonstrate the accuracy of the proposed tracking algorithm and its robustness to the absence of the labeled data thanks to the artificial labeled data.

## 1. Introduction

Wireless sensor networks (WSNs) consist of small, low-power sensor nodes that collect, process, and communicate the sensor data. WSNs are suitable to monitor environmental phenomena widely distributed in space and time. This paper focuses on the problem of localization and classification of multiple targets moving within the area of deployed sensor nodes, which plays an important role in surveillance application [1], environmental monitoring [2–4], and traffic monitoring [5].

**Figure 1.**
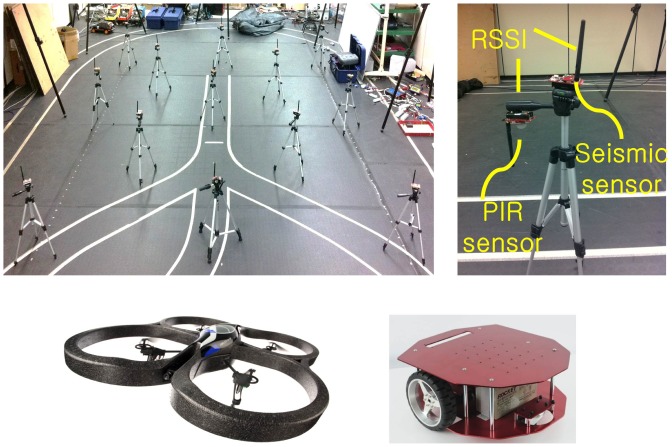
Experimental setup (clockwise): 4 m × 4 m size surveillance area, deployed sensor nodes on a tripod, ground and aerial targets.

In particular, this paper considers low-cost sensors such as acoustic, magnetic, seismic, ultrasonic sensors and radio signal strength as shown in Figure 1, which consume low power. The low-cost sensors limit the direct adaptation of existing multi-target tracking algorithm due to following reasons. First, many multi-target tracking algorithms, such as Gaussian mixture probability hypothesis density filter [6], Markov chain Monte Carlo data association [7,8], assume that sensors measure location or both range and angle of targets, for example by using RFID [9], sonar, and LiDAR [10]. Generally, low-cost sensors such as RSSI measure relative distance to the target, not location. Therefore, without employing some additional data-association algorithms, it is difficult to directly adopt those algorithms [6–10] in our setup. Second, popular conventional localization algorithms such as time of arrival (TOA), time difference of arrival (TDOA) and angle of arrival (AOA) need expensive hardware [11,12] (e.g., laser) that is not available for the low-cost sensor nodes. Third, the memory and computational power in the low-cost sensor network are limited. There are existing classification methods such as fast Fourier transform (FFT) [13,14] and feature-aided tracking [15–17] in order to extract feature points from raw data. These need heavy computational load and large memory, which are intractable for low-cost sensor nodes without additional digital signal co-processors.

**Figure 2.**
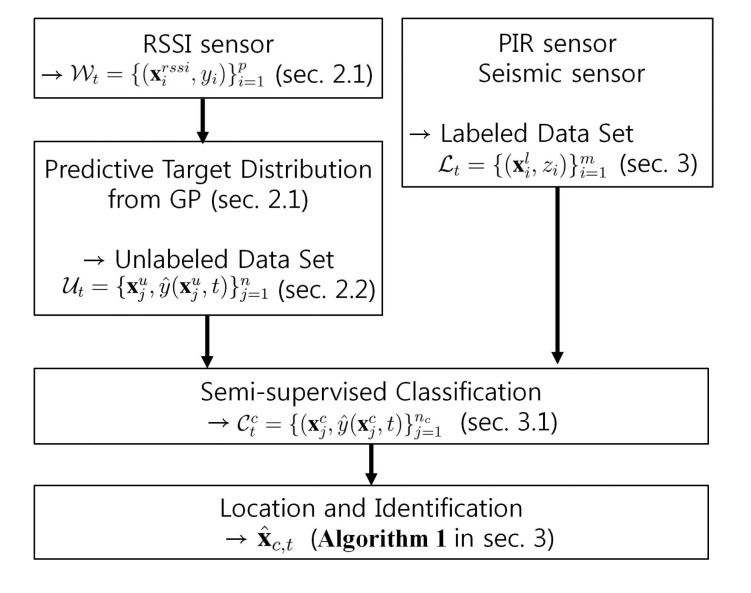
Overview of the localization and classification algorithm.

In order to meet the requirement of the multi-target tracking using low-cost sensor networks, we propose a new tracking algorithm based on semi-supervised learning as described in Figure 2. When a few labeled data are available, the semi-supervised learning [18] can efficiently utilize large amount of unlabeled data. In our experimental scenario, labeled data are obtained by deployed passive infrared (PIR) sensors and seismic sensors. This setup is suitable for semi-supervised learning because labeled data are sparsely obtained over space from those sensors due to their narrow sensing range relative to the wide surveillance area.

While labeled data present type information of heterogeneous targets, unlabeled data contain information about locations of targets. In the proposed framework, unlabeled data are generated from predictive target distribution that represents the probability of predicted target locations. To obtain the distribution from RSSI measurements, we employ Gaussian process (GP), which is a non-parametric machine learning technique. Due to its flexibility in modeling complex expressions using a small number of parameters, GP has been widely used to approximate signal strength [19].

Unlabeled data generated by GP contain information of target locations. Then, unlabeled data are classified via semi-supervised learning along with the small amount of labeled data, which provide information of both locations and identifications of targets. In other words, we can track targets using GP and semi-supervised learning in sequence.

We also propose a technique to deal with absence of labeled data, which can occur due to sensors fault, lack of the deployed sensors, or communication failure. We overcome this problem by propagating support vectors of each time instant, *i.e.*, essential data points during classification, to the next time step and denning those that match with the incoming unlabeled data as artificial labeled data. This allows to maintain target tracking regardless of the existence of the labeled data.

The proposed algorithm is applied to an experiment for the tracking of moving aerial and ground vehicles, as shown in Figure 1. From the experiments with an actual wireless sensor network in a 4 m × 4 m surveillance area, our algorithm achieves good localization results. Moreover, the suggested algorithm is robust to the absence of the labeled data thanks to the artificial labeled data.

The rest of this paper is organized as follows. Section 2 introduces RSSI-based predictive target distribution using a Gaussian process. In Section 3, the tracking algorithm using semi-supervised learning is described. Section 4 shows the experimental results of tracking aerial and ground targets. Finally, Section 5 is devoted to concluding remarks.

## 2. RSSI-Based Predictive Target Distribution

**Figure 3.**
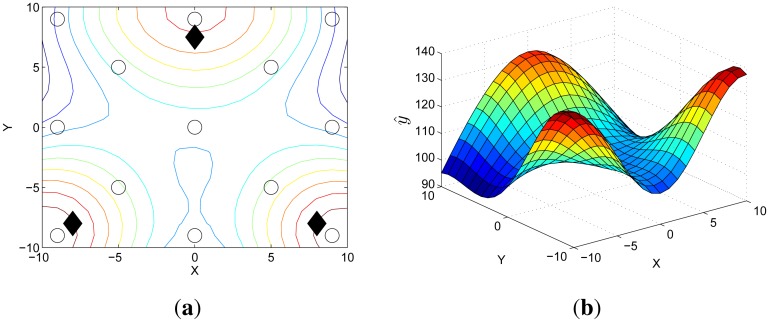
Predictive target distribution is described where (**a**) deployed sensor nodes (circles) measure the RSSIs emitted from the three targets (diamonds) and these measurements are used to make the distribution; and (**b**) the 3-D distribution using a Gaussian process is shown. In the RSSI-based distribution, the higher value on the *z*-axis denotes higher probability of the predicted locations of the targets.

In received signal strength indication (RSSI)-based localization, the signal strength emitted by targets is measured by many sensors spatially distributed near the targets [20]. With the known locations of sensors, these measurements are used to make predictive target distribution that represents the probability of the predicted target locations. Figure 3 is a distribution example where 13 sensor nodes measure the RSSIs emitted from three targets.

Section 2.1 describes how the predictive target distribution is obtained by using a Gaussian process (GP). Section 2.2 shows how unlabeled data are generated from the constructed predictive target distribution.

### 2.1. Gaussian Process

Gaussian process for regression (GP) is a non-parametric regression model that defines a Gaussian distribution over output value for input value, conditioned on training data. The advantage of GP is its flexibility in modeling complex expressions under noise using a small number of learning parameters.

Let *y*_1_,…,*y_p_* be the measurement set where *y_i_* is the RSSI measurement obtained from the target nearest to the *i^th^* node and *p* is the number of RSSI sensor nodes. Also, sensor locations are defined as ${\text{x}}_{1}^{\text{rssi}}$,…, ${\text{x}}_{p}^{\text{rssi}}$. Then, $\text{W}={\left\{\left({\text{x}}_{i}^{\text{rssi}},y_{i}\right)\right\}}_{i=1}^{p}$ is the training set obtained from

(1) $$y_{i}=g\left({\text{x}}_{i}^{\text{rssi}}\right)+ɛ$$

where the Gaussian noise *ε* is drawn from normal distribution $N(0,\sigma_{gp}^{2})$. In the GP model, the joint distribution over the noisy training outputs 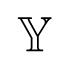 = {*y*_1_,…,*y*_p_}*^T^* is a function of the training inputs $\text{X}=\left\{{\text{x}}_{1}^{\text{rssi}},\ldots ,{\text{x}}_{p}^{\text{rssi}}\right\}$, with the form

(2) $$\text{Y}∼\text{N}\left(0,K\left(\text{X},\text{X}\right)+\sigma_{gp}^{2}I\right),$$

where *K*( 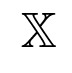), ( 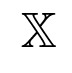) is *p* × *p* kernel matrix whose (*i, j*)*^th^* element is *k*(**x***_i_*,**x***_j_*). The squared exponential is a commonly used kernel function, given by

(3) $$k\left({\text{x}}_{i},{\text{x}}_{j}\right)=\theta_{1}\exp\left(\frac{-{\left\|{\text{x}}_{i}-{\text{x}}_{j}\right\|}^{2}}{2\theta_{2}}\right)$$

where *θ*_1_ and *θ*_2_ are hyperparameters of the kernel.

After the training, the GP estimates the output value of the Gaussian process for the input value that is an interested location x_*_ = [X, Y]^*T*^ ∈ χ, where χ is a user-defined set of the interested location and its cardinality is *υ* = |χ|. The output value has the mean *ŷ*(x_*_) and variance *σ̂*^2^(x_*_):

(4) $$g\left({\text{x}}_{*}\right)∼N\left(ŷ\left({\text{x}}_{*}\right),{\hat{\sigma }}^{2}\left({\text{x}}_{*}\right)\right)$$

where

$$\begin{array}{ll} ŷ\left({\text{x}}_{*}\right) & =K{\left({\text{x}}_{*},\text{X}\right)}^{T}{\left(K\left(\text{X},\text{X}\right)+\sigma_{GP}^{2}I\right)}^{-1}\text{Y}\\ {\hat{\sigma }}^{2}\left({\text{x}}_{*}\right) & =k\left({\text{x}}_{*},{\text{x}}_{*}\right)-K{\left({\text{x}}_{*},\text{X}\right)}^{T}{\left(K\left(\text{X},\text{X}\right)+\sigma_{GP}^{2}I\right)}^{-1}K\left({\text{x}}_{*},\text{X}\right) \end{array}$$

We note that *ŷ*(x_*_,*t*) represents the GP estimation at time step *t.*

#### Example 1

*Figure 3b is an example of the distribution obtained by the GP where χ is defined as* {(X, Y) : X, Y ∈ {−10,…,10}} and the values on the z-axis are the estimated means. When using the RSSI measurements as the input of the GP and the corresponding locations as the output of the GP, the distribution represents the predicted sensor measurements over the space as illustrated in Figure 3b. More specifically, the estimated ŷ(x_*_) indicates the predicted RSSI measurement of an artificial sensor that does not exist at x_*_. Because the RSSI value is larger when the target is closer, the value on the z-axis of the distribution, ŷ, implies the probability with which the targets are located at (X, Y).

### 2.2. Unlabeled Data Generation

We highlight again that larger value on the *z*-axis of the predictive target distribution denotes higher probability of the predicted locations of targets. In this manner, we derive a dataset ${\text{U}}_{t}={\left\{\left({\text{x}}_{j}^{u},ŷ\left({\text{x}}_{j}^{u},t\right)\right)\right\}}_{j=1}^{n}$ for **x***^u^* ∈ **x** from the predictive target distribution satisfying $ŷ\left({\text{x}}_{j}^{u},t\right)\geq y_{th}$ where *y_th_* is a user-defined threshold. Thus, the dataset 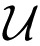
*_t_* are correlated with the predictive locations of the targets at time *t.* Depending on the value of *y_th_*, the amount of unlabeled data is decided. As described in Figure 3b, the value larger than 130 can be considered high probability for the target locations. For this reason, we set *y_th_* = 130 in this paper.

We assume that sensor nodes do not know the labels of the RSSI measurements, *i.e.,* the nodes do not know who emitted the RF signal. With this assumption, the dataset 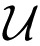
*_t_* does not provide any information about identity of the targets. In the following section, we introduce a semi-supervised algorithm to classify the unlabeled data in order to identify the targets.

## 3. Target Tracking Based on Semi-Supervised Learning

Traditional classification algorithms such as supervised learning are inaccurate when only a few labeled data are available. To overcome this problem, semi-supervised classification [21,22] improves the efficiency and performance of supervised learning by using both labeled and unlabeled data.

We assume that *N* different targets are identified by the identification label set {1,…, *N*}. There is a small set of labeled data, given by ${\text{L}}_{t}={\left\{\left({\text{x}}_{i}^{l},z_{i}\left({\text{x}}_{i}^{l},t\right)\right)\right\}}_{i=1}^{m}$, where ${\text{x}}_{i}^{l}$ is the location of the *i^th^* labeled data and $z_{i}\left({\text{x}}_{i}^{l},t\right)$ is the observation denoting the identification label of the targets.

We describe the Laplacian support vector machine (LapSVM) algorithm in Section 3.1. The best scenario for semi-supervised classification is where there always exists at least one labeled data for each class while tracking the target. However, this cannot be guaranteed due to sensor fault, lack of deployed sensor, or communication failure. Considering a case where there is no labeled data at some time instants, we propose incremental learning for target tracking in Section 3.2.

### 3.1. Laplacian Support Vector Machine (LapSVM)

In the graph-based semi-supervised classification algorithm, the geometry of data is represented by an undirected graph 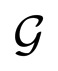 = (*V, E*) with nodes *V* = {1,…, *n* + *m*} and edges *E,* where *n* and *m* are the number of unlabeled and labeled data, respectively. The nodes *V* represent the training data, and the edges *E* denote the similarities between the nodes. These similarities are given by the connection strength from a node *i* to another node *j,* which is encoded in the element *W_ij_*, of a weight matrix *W*. A typical weight matrix is given by the a Gaussian function of the Euclidean distance between data, with length scale *θ*_3_:

(5) $$W_{ij}=\{\begin{array}{ll} \exp\left(\frac{-{\left\|{\text{x}}_{i}-{\text{x}}_{j}\right\|}^{2}}{\theta_{3}}\right) & \text{if}\left(i,j\right)\in E\\ 0 & \text{otherwise} \end{array}$$

where training dataset $\left\{{\text{x}}_{1},{\text{x}}_{1},\ldots ,{\text{x}}_{n+m}\right\}=\left\{{\text{x}}_{1}^{u},\ldots ,{\text{x}}_{n}^{u},{\text{x}}_{1}^{l},\ldots ,{\text{x}}_{m}^{l}\right\}$ consists of unlabeled and labeled data points. An edge (*i,j*) between the nodes **x***_i_* and **x***_j_* is linked by the *k* nearest neighbors. To assign the weights to the edge set of 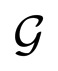, the graph Laplacian matrix is defined as

(6) L=D−W

where *D* is a diagonal matrix with entries $D_{ii}=\sum_{j=1}^{n+m}W_{ij}$.

The semi-supervised learning algorithm outputs an (*n* + *m)*-dimensional real-valued vector f* = [*f*(**x**_1_),*f*(**x**_2_),…,*f*(**x***_n_*_+_*_m_*)]*^T^*, where $f\left({\text{x}}_{j}\right)=\sum_{i=1}^{n+m}\alpha_{i}^{*}k\left({\text{x}}_{i},{\text{x}}_{j}\right)$. In the LapSVM algorithm, optimal α* = [*α*_1_,…,*α_n+m_*]*^T^* is given by

(7) $$\alpha^{*}={\left(2\gamma_{A}I+2\frac{\gamma_{I}}{{\left(n+m\right)}^{2}}LK\right)}^{-1}J^{T}Z^{\left(l\right)}\beta^{*}$$

where *Z*^(^*^l^*^)^ is an *m*-by-*m* diagonal matrix given by $Z_{ii}^{\left(l\right)}=z_{i}$with *z_i_* ∈ { −1,+1} and *J* is an *m*-by-(*m* + *n*) matrix given by [*I_m_,_m_* *O_m_,_n_*] where *I_m,m_* is an *m*-by-*m* identity matrix and *O_m_,_n_* is an *m*-by-*n* rectangular matrix with all zeros. Tuning variables *γ_A_* and *γ_I_* are weight parameters, and *K* is the kernel matrix defined in Equation (3).

The optimal β* in order to get the optimal α* in Equation (7) is obtained by the following quadratic program:

(8) $$\beta^{*}=\underset{\beta \in R^{m}}{\max}\sum_{i=1}^{m}\beta_{i}-\frac{1}{2}\beta^{T}Q\beta$$

subject to:

(9) $$\sum_{i=1}^{m}z_{i}\beta_{i}=0$$

(10) $$0\leq \beta_{i}\leq \frac{1}{m}\text{for}i=1,\ldots ,m,$$

where

(11) $$Q=Z^{\left(l\right)}JK{\left(2\gamma_{A}I+2\frac{\gamma_{I}}{{\left(n+m\right)}^{2}}LK\right)}^{-1}J^{T}Z^{\left(l\right)}$$

In the semi-supervised learning, the unlabeled data help finding the decision boundary more accurately, assuming that data points of each class tend to form a cluster (called cluster assumption [21]). In other words, if points are in the same cluster, they are likely to belong to the same class.

#### Example 2

**Figure 4.**
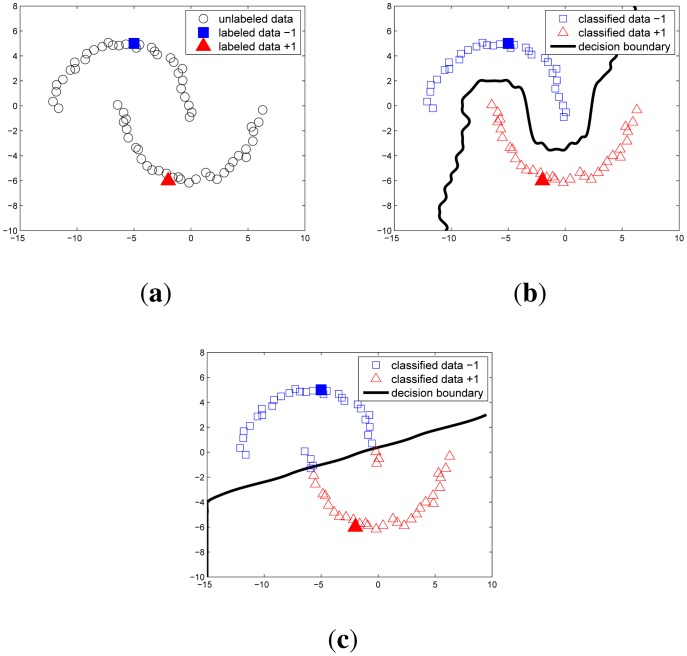
Two moon data classification where (**a**) 64 unlabeled and 2 labeled data before classification; (**b**) result of the LapSVM semi-supervised learning; (**c**) result of the standard SVM.

*Figure 4 illustrates the two-moon classification problem. In order to show the usefulness of unlabeled data, we prepare only one labeled data for each class. In the semi-supervised algorithm, the adjacent points in a high-density data region are classified into the same class. This gives a correct decision boundary, in contrast to the supervised learning shown in Figure 4c. The influence of the unlabeled data in this algorithm is handled by γ_I_ in* *Equation* (7)*. When γ_I_* = 0, *Equation* (7) *gives zero coefficients over the unlabeled data, so the algorithm becomes standard supervised SVM. On the other hand, as γ_I_ increases, the effect of unlabeled data also increases. In this paper, we set γ_I_* = 10^3^ *and γ_A_* = 0.1.

General classification algorithm presets the label value to +1 or −1 and appoints the label of an unlabeled data **x***^u^* based on the function *f* : if *f*(**x***^u^*) > 0 then the label of **x***^u^* is +1, or if *f*(**x***^u^*) ≤ 0 then the label of **x***^u^* is −1. For the multi-label classification problem, it can be solved using “One Against One” or “One Against All” algorithms [23]. Specific descriptions of these algorithms are out of the scope of this paper. Instead, we define *Q*(**x**) as a function mapping the data to the label. Therefore, all data points ${\text{x}}_{i}^{u}$, 1 ≤ *i* ≤ *n* will have labels through the classifier, given by $Q\left({\text{x}}_{i}^{u}\right)\in \left\{1,\ldots ,N\right\}$, where *N* is the number of identification labels. For example, we consider two types of targets to track, thus *N* = 2.

As the result of classification, we define ${\text{C}}_{t}^{c}$ as classified sets to label c, given by ${\text{C}}_{t}^{c}={\left\{({\text{x}}_{j}^{c},ŷ\left({\text{x}}_{j}^{c},t\right)|Q\left({\text{x}}_{j}^{c}\right)=c\right\}}_{j=1}^{n_{c}}\text{for}c\in \left\{1,\ldots ,N\right\}$, where *n_c_* is the number of data points that are labeled to *c*. The total set and number of unlabeled data, 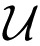
*_t_* and *n* described in Section 2.2, can be described by $n=\sum_{c=1}^{N}n_{c}$ and $U_{t}=\cup_{c=1}^{N}C_{t}^{c}$.

### 3.2. Incremental Time Series Classification

We consider a situation where there is no labeled data after the targets intrude the surveillance field. Most papers assume that initial positions of the intruders are successfully estimated by deploying sensor nodes intensively at edge of the surveillance field. Accordingly, we also assume that the set of labeled data exists at the moment the targets just enter the surveillance area. In this section, we focus on how to perform semi-supervised classification when labeled data is absent after the target intruded.

Target information at the current time step is correlated with the information at the previous time step such as a Markov process model. This leads us to exploit the classified data at the previous time step to create artificial labeled data for the current time step (artificial labeled data is different with the genuine labeled data made by the sensor). Our strategy is to select useful data among the classified datasets at the previous time step and define them as the artificial labeled data for the current time step.

The support vector machine framework offers a natural method to select such useful data. The classification rule is based on the decision boundary that depends on the support vector, *x_i_* with |*α_i_*| < 0 in Equation (7), in the SVM algorithm. In other words, the support vectors are representative points of the classification result. Moreover, once the data is well trained, most of the *α*'s become zero and only a few support vectors have nonzero value of the coefficient |*α*|. Therefore, the small set of support vectors is suitable to be propagated to the next step and they become candidates for artificial labeled data.

One more consideration in constructing the artificial labeled data is that they have to be updated as time elapses. Some candidates obtained during the previous time step may not correlate with the current data. In order to find correlated points among the candidates to the current data, we check whether the candidates belong to the current unlabeled dataset or not. The candidates satisfying this condition become the artificial labeled data at time *t,* given by ${\text{A}}_{t}^{c}={\left\{\left({\text{x}}_{i}^{c},z_{i}\left({\text{x}}_{i}^{c},t-1\right)\right)|\left|\alpha_{i}\right|>0,{\text{x}}_{i}^{c}\in {\text{C}}_{t}^{c}\cap {\text{C}}_{t-1}^{c}\right\}}_{i=1}^{s_{c}}$ where *s_c_* is the number of the artificial labeled data that are labeled to *c*.

#### Example 3

**Figure 5.**
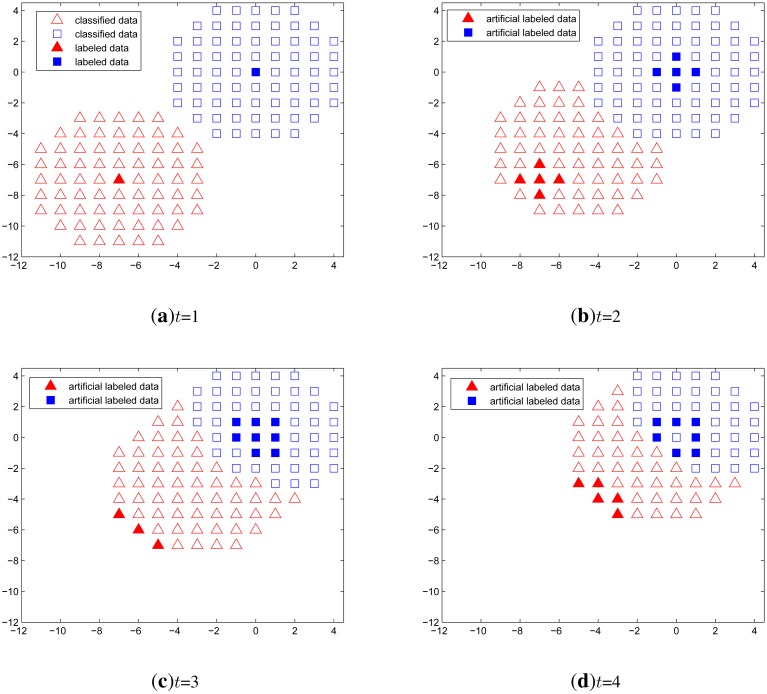
Time series classification when one target approaches the other target at the origin. Unlabeled data is generated over a circular region around the two targets, as marked by blank triangle and square symbols. Among those data, classified points are denoted as filled triangle and square. At the time instant shown in (**a**), it is assumed that labeled data exists for each target. After that, labeled data is not available from (**b**) to (**d**), so semi-supervised classification is performed using only the artificial labeled data.

Figure 5 shows a time series classification example when no labeled data exist except in the first time step. In this simulation, we let one target remain at the origin all the time and the other approaches the origin. The unlabeled data are generated over a circular region around the two targets. The expected incorrect outcome is that one classified set is merged into the other set, which wrongly represents multiple overlapping targets as one identity. However, from the simulation results, the classification using only the artificial labeled data performs well both when the targets are approaching and when the targets are apart from each other.

Concluding this section, we summarize a procedure for multi-target localization and classification into Algorithm 1.


**Algorithm 1** Multi-target localization and classification.
 **Step 1:** Collect *p* RSSI measurement dataset 
${\text{W}}_{t}={\left\{\left({\text{x}}_{i}^{\text{rssi}},y_{i}\right)\right\}}_{i=1}^{p}$ **Step 2:** Collect *m* labeled dataset 
${\text{L}}_{t}={\left\{\left({\text{x}}_{i}^{l},z_{i}\right)\right\}}_{i=1}^{m}$ **Step 3:** Generate the predictive target distribution (Section 2.1) **Step 4:** Obtain unlabeled dataset from the predictive target distribution 
${\text{U}}_{t}={\left\{{\text{x}}_{j}^{u},ŷ\left({\text{x}}_{j}^{u},t\right)\right\}}_{j=1}^{n}$. (Section 2.2) **Step 5:** If *t* ≥ 2, generate artificial labeled data 
${\text{A}}_{t}^{c}$, then renew 
${\text{L}}_{t}={\text{L}}_{t}\cup {\text{A}}_{t}^{c}$. (Section 3.2) **Step 6:** Execute the LapSVM classification algorithm, then obtain the classified dataset 
${\text{C}}_{t}^{c}={\left\{({\text{x}}_{j}^{c},ŷ\left({\text{x}}_{j}^{c},t\right)\right\}}_{j=1}^{n_{c}}\text{for}c\in \left\{1,\ldots ,N\right\}$. (Section 3.1) **Step 7:** Estimate the positions of the targets **x̂***_c,t_* for *c* = {1,…,*N}*:   
${\hat{\text{x}}}_{c,t}={\text{x}}_{k}^{c}$,   
$k=\arg\max_{j}ŷ\left({\text{x}}_{j}^{c},t\right)$,  where 
$ŷ\left({\text{x}}_{j}^{c},t\right)\in C_{t}^{c}\text{for}j\in \left\{1,\ldots ,n_{c}\right\}$. **Step 8:**
*t*←*t* + 1, then go to **Step1**.


## 4. Experiment

This section describes experimental setup, characteristics of sensor data, (* i.e.*, PIR, seismic, RSSI sensors), and tracking results.

### 4.1. Setup

We consider a two-dimensional tracking problem for one ground and one aerial target using a total of 26 sensor nodes. To identify the aerial vehicle, 13 spatially distributed seismic sensors are used to recognize the airflow due to aerial vehicle. The ground vehicle is identified by 13 passive infrared sensors (PIR). In addition, RSSI is available for both types of sensor nodes. The true trajectories of the targets are obtained by a Vicon Motion System that tracks reflective markers attached to each target.

### 4.2. Characteristics of Sensor Data

**Figure 6.**
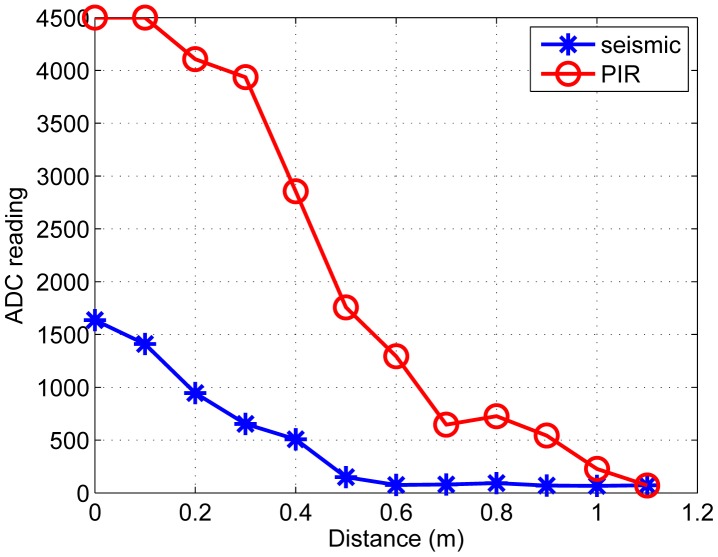
Raw ADC readings of PIR sensor with respect to the distance between the PIR node and ground target and seismic sensor with respect to the distance between the seismic node and aerial target.

We record the raw ADC readings of one PIR sensor by varying the distance between the node and the ground target. Similarly, the raw ADC of one seismic sensor is measured using the aerial target, shown in Figure 6. We set threshold values to each sensor, *i.e.*, 300 for the seismic sensor and 500 for the PIR sensor. Based on the defined thresholds on readings, the *i^th^* seismic sensor node yields +1 labeled data when a reading is greater than the threshold, *i.e., z_i_* = +1. Similarly, the *j^th^* PIR sensor node generates −1 labeled data, *i.e., z_j_* = −1.

**Figure 7.**
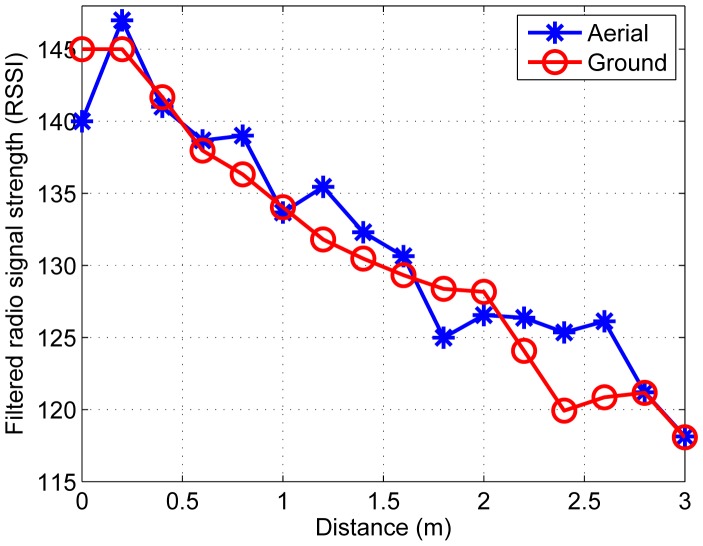
RSSI measurements adopting moving average filter with respect to the distance between a node and the ground target and the distance between a node and the aerial target.

Besides, all 26 sensor nodes measure the RSSI. Considering the noisy ADC reading of the RSSI, moving average filter [24] is adopted. Figure 7 shows the relationship between the filtered RSSI measurement and the distance between a node and the ground target and the aerial target, respectively.

### 4.3. Tracking Results

**Figure 8.**
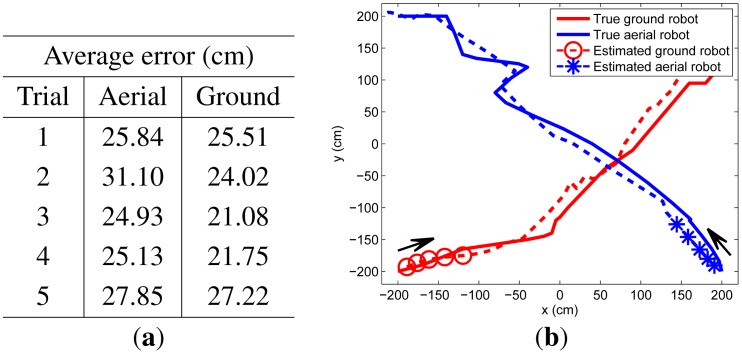
Five experimental tracking results for moving ground and aerial targets with (a) average error and (b) true and estimated trajectories of the first trial experiment.

In our experiments, two targets start from different corners of the surveillance area, crossing at the middle of their pathway. We perform five trials, whose results are summarized in Figure 8. Because the RSSI characteristics are affected by the motion of the flying aerial robot, its tracking results are worse than results of the ground robot.

Moreover, we analyze the effect of the artificial labeled data introduced in Section 3.2 with respect to the ratio of existence of the labeled data, R:

(12) $$\text{R}=\frac{\sum_{i}L\left(t_{i}\right)}{T}\times 100$$

(13) $$L\left(t_{i}\right)=\{\begin{array}{cc} 1, & \text{if the labeled data is present at}\;t_{i}\\ 0, & \text{if the labeled data is absent at}\;t_{i}, \end{array}$$

where *t_i_* is discrete time step and *T* is total tracking time.

**Figure 9.**
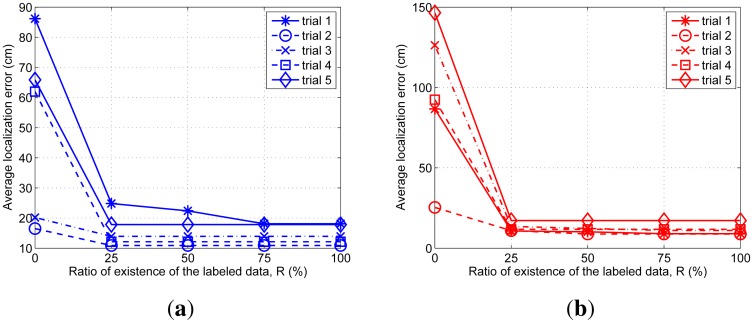
Average localization error from five experiments according to the existence ratio of the labeled data, (**a**) Localization error of the aerial target; (**b**) Localization error of the ground target.

We intentionally remove the labeled data among the data obtained via the experiment up to each 25%, 50%, 75% 100%, and then perform tracking. The results of the average localization error for each aerial and ground targets are summarized in Figure 9.

When there is no labeled data all the time (R = 0%), four of the five results do not indicate the correct trajectories. From 25% to 100% of the existence ratio of the labeled data, the results are almost same as the results for 100% absence ratio. We confirm that the artificial labeled data prevent the tracking performance from getting deteriorated by the lack of the labeled data.

## 5. Conclusions

This paper proposed a new multi-target localization and classification for low-cost sensor networks. Data configuration that consists of the unlabeled and labeled data obtained from low-cost sensor nodes was well-suited to apply semi-supervised learning. Furthermore, the incremental algorithm was developed with semi-supervised learning to handle the situation where labeled data are absent. The suggested algorithm was evaluated for the tracking of aerial and ground targets and showed accurate tracking performance and robustness to the absence of labeled data.
